# Redox-Responsive GHK-Conjugated Sponge Spicules for Sustained Dermal Delivery and Enhanced Collagen Synthesis

**DOI:** 10.3390/mi17060750

**Published:** 2026-06-21

**Authors:** Won-Kyu Hong, Patrick Po-Han Huang, Diane Duncan, Rocha Marco, Ho-Sung Choi, Young-Wook Jo

**Affiliations:** 1 Department of Biotechnology, Yonsei University, Seoul 03722, Republic of Korea; hwk0417@gmail.com (W.-K.H.);; 2 Class One Clinic, Seoul 06232, Republic of Korea; 3 Surgery and Aesthetics, Huang PH Dermatology, Kaohsiung 804, Taiwan; 4 Department of Surgery, University of Colorado Health, Aurora, CO 80045, USA; 5 Departamento de Dermatologia, Federal University of São Paulo, São Paulo 04039-001, Brazil; marcoderm@hotmail.com; 6 LIEUL Seoul Clinic, Seoul 06035, Republic of Korea

**Keywords:** ALTUM, functionalized sponge spicules, redox-responsive release, GHK peptide

## Abstract

Sponge spicules have emerged as promising biomaterial scaffolds due to their biocompatibility and unique structural properties; however, achieving stable and bioactive functionalization remains a key challenge. The tripeptide GHK is known to promote collagen synthesis and wound repair, yet its therapeutic efficacy is often limited by rapid diffusion and instability. Here, we report ALTUM, a thiol-functionalized sponge spicule composite in which GHK is covalently conjugated via disulfide linkage to enable controlled and redox-responsive peptide delivery. ALTUM exhibited sustained GHK retention under physiological and storage conditions, while exposure to reduced glutathione (GSH) selectively accelerated peptide release through disulfide bond cleavage. This dual release behavior—long-term stability combined with reduction-triggered activation—distinguishes ALTUM from conventional delivery systems. The composite also demonstrated structural stability under thermal, cyclic, and photostability conditions. In an artificial human skin model, ALTUM enhanced dermal penetration of GHK and significantly increased collagen deposition in the dermal layer, demonstrating its capacity to promote collagen production within deeper skin tissue, compared to simple spicule–peptide mixtures. ALTUM was fabricated at an optimized spicule-to-peptide ratio of 3% (*w*/*w*), preserving the needle-shaped spicule morphology after surface modification. In vitro, ALTUM exhibited a sustained release profile, with GHK release markedly accelerated in the presence of 10 mM glutathione (GSH) compared with non-reductive conditions, reaching approximately 60% cumulative release over 35 days. In the bioprinted artificial human skin model, ALTUM delivered 9.72 ng/cm^2^ of GHK, more than five-fold higher than the physical mixture of spicules and free GHK (1.9 ng/cm^2^), and significantly increased type I collagen expression in human dermal fibroblasts. Mechanistically, ALTUM-mediated delivery was associated with increased TGF-β expression and engagement of the SMAD signaling pathway, as indicated by increased phosphorylation of SMAD2/3, consistent with involvement of the TGF-β–SMAD axis in the observed collagen induction. Collectively, these findings establish ALTUM as a structurally stable, redox-responsive dermal delivery platform that enhances collagen synthesis and skin regeneration.

## 1. Introduction

Collagen is a fundamental component of the extracellular matrix (ECM), playing a crucial role in maintaining skin structure, elasticity, and overall integrity [1]. All types of collagens comprise 70–80% of the dermal ECM; these act as the main load bearing structural proteins of the skin [2]. As the primary structural protein in the dermis, collagen is essential for skin regeneration and wound healing. However, collagen production declines with age, leading to skin aging characterized by wrinkles, reduced elasticity, and impaired healing capacity [3]. Therefore, enhancing collagen synthesis has become a key therapeutic target in anti-aging treatments and tissue regeneration strategies. Effective delivery systems that can promote sustained collagen production are critical for addressing these challenges and improving skin health over the long term.

Sponge spicules, the skeletal elements of marine sponges, have recently gained attention in tissue engineering due to their biocompatibility, unique structural properties, and potential for functionalization [4]. Their porous nature and high surface area make them excellent candidates for supporting cell growth and tissue integration. Recent studies have explored the use of sponge spicules as scaffolds for bone regeneration, drug delivery, and as vehicles for bioactive molecules [5]. However, while these applications have shown promise, the challenge remains to optimize the functionalization of sponge spicules to enhance their bioactivity and stability in therapeutic contexts.

The functionalization of sponge spicules has been an area of active research, with various methods being explored to improve their biological functionality. Previous studies have primarily focused on modifying the surface of silicate nanoparticles with chemical groups or bioactive agents to enhance their interaction with cells and tissues [6]. For instance, functionalization with amine or carboxyl groups has been shown to improve cell adhesion and proliferation [7]. However, these approaches often result in limited bioactivity and stability of the functionalized spicules, particularly under physiological conditions. The challenge remains to develop functionalization strategies that not only stabilize the bioactive molecules on the spicule surface but also enable their sustained release.

The tripeptide GHK (Glycyl-L-Histidyl-L-Lysine) is widely recognized for its ability to promote collagen synthesis and wound healing [8]. GHK has been shown to influence a variety of biological processes, including cell proliferation, anti-inflammatory responses, and ECM remodeling [9]. Despite its well-documented bioactivity, the exact mechanisms through which GHK enhances collagen synthesis remain unclear.

This study seeks to establish a novel functionalization strategy for sponge spicules through the covalent conjugation of GHK-peptide to sulfhydryl-functionalized spicules, thereby creating ALTUM, a stable and bioactive composite. The name ALTUM derives from the Latin *altum* (“deep”), denoting the platform’s design objective of delivering peptides into deeper dermal layers through spicule-mediated mechanical penetration combined with sustained, redox-responsive release. Structurally, ALTUM consists of needle-shaped silica-based spicules isolated from *Spongilla fragilis* whose surface silanol groups are converted to sulfhydryl (–SH) functionalities via silanization with (3-mercaptopropyl)trimethoxysilane (MPTMS), generating SP-SH. The thiolated spicule surface is then covalently coupled to a cysteine-bearing GHK peptide (HS-GHK) through thiol–disulfide exchange, yielding a disulfide (S–S) tether between the spicule and the GHK moiety. The primary objective is to assess the efficacy of ALTUM in facilitating sustained collagen synthesis and elucidating its underlying mechanisms. By systematically investigating the structural stability, release kinetics, and bioactivity of ALTUM, with particular emphasis on its capacity to enhance collagen production via the sustained release of GHK-peptide and effective penetration into deeper skin layers, this research aims to advance the development of functionalized biomaterials for therapeutic applications.

## 2. Materials and Methods

### 2.1. Preparation and Functionalization of ALTUM

Natural spicules from *Spongilla fragilis* were purified using NaOH and HCl treatments. Sulfhydryl groups were introduced via silanization with MPTMS (70 °C, 5 h), followed by covalent conjugation with HS-GHK peptide (room temperature, 10 h) to synthesize ALTUM.

### 2.2. Physicochemical Characterization

The surface morphology and elemental composition of SP and ALTUM were analyzed using SEM/TEM, EDS, and XPS (K-Alpha+, Thermo Fisher Scientific, East Grinstead, UK). Surface-accessible thiols were quantified using Ellman’s reagent (DTNB).

### 2.3. Sulfhydryl Functionalization and Peptide Conjugation

To purify and functionalize the needle-shaped spicules derived from *Spongilla fragilis*, 300 g of spicules were first mixed with 3 L of water for 10 min. The mixture was then separated into smaller portions, and impurities and broken spicules were removed using sieves and running water. After this initial purification, the spicules were washed with water and dried at 70 °C. To remove organic matter, the dried spicules were mixed with water, and 50 g of NaOH was added. This mixture was allowed to react for 2 h, after which the supernatant was discarded. The spicules were then mixed with water and stirred for 1 h. This washing process was repeated five times. The resulting mixture was filtered, washed with water, and dried again at 70 °C. To further remove impurities, 100 g of the dried spicules were mixed with 500 mL of water and 40 mL of hydrochloric acid and reacted at 70 °C for 6 h. The mixture was then filtered, washed with water, and dried at 70 °C. To introduce sulfhydryl (-SH) groups, 60 g of the purified spicules were mixed with toluene, and 0.5 mL of (3-Mercaptopropyl)trimethoxysilane was added. The reaction was carried out at 70 °C for 5 h. After the reaction, the spicules were washed with toluene, acetone, and water. The washed spicules were then mixed with 400 mL of water and 10 mL of hydrochloric acid and reacted for 30 min. The mixture was filtered, washed with water, and dried at 70 °C. Finally, 30 g of the sulfhydryl-modified spicules were mixed with a buffer solution and 1 g of HS-GHK peptide, and the reaction was allowed to proceed at room temperature for 10 h. The mixture was filtered, washed with water, and dried at 70 °C for at least 48 h to form the sulfhydryl-tripeptide conjugate. The GHK peptide was used as a synthetically pre-thiolated, cysteine-bearing derivative (HS-GHK) carrying a free terminal thiol group (obtained by solid-phase peptide synthesis); no additional thiolation of the peptide was performed in-house. Conjugation thus proceeds through thiol–disulfide exchange between the surface –SH groups of SP-SH and the free thiol of HS-GHK, generating a covalent disulfide (S–S) linkage between the spicule surface and the peptide.

### 2.4. X-Ray Photoelectron Spectroscopy (XPS)

Native sponge spicules (SP), thiol-functionalized spicules (SP-SH), and GHK-conjugated thiolated spicules (ALTUM) were dried under vacuum (≥12 h) prior to analysis to minimize physisorbed water. Dried powders were immobilized onto conductive carbon tape (PELCO Tabs™, Ted Pella, Ted Pella, Inc., Redding, CA, USA) mounted on stainless-steel sample stubs. To reduce sample charging, conductive grounding was ensured by pressing the sample gently into the tape and connecting the tape edge to the stub. All samples were prepared in the same manner and analyzed at room temperature.

XPS measurements were performed using an X-ray photoelectron spectrometer equipped with a monochromated Al Kα X-ray source (hν = 1486.6 eV) (K-Alpha+, Thermo Fisher Scientific, East Grinstead, UK) under ultrahigh vacuum conditions (base pressure < 1 × 10^−9^ mbar). Survey spectra were acquired over a wide binding energy range (typically 0–1200 eV) using a pass energy of 150 eV and an energy step size of 1.0 eV. High-resolution spectra for S 2p were acquired using a pass energy of 20–40 eV and an energy step size of 0.05–0.1 eV. Charge compensation was applied using a low-energy electron flood gun when needed.

All spectra were charge-corrected by referencing the C 1s peak of adventitious carbon to 284.8 eV. Data processing and peak fitting were performed using commercial software (Avantage Version 6, Thermo Fisher Scientific, UK) or equivalent (CasaXPS Version 2.3, Casa Software Ltd., Devon, UK). Atomic percentages from survey spectra were calculated using manufacturer-provided sensitivity factors after background subtraction.

For S 2p peak deconvolution, a Shirley-type background was applied to the high-resolution spectrum. The S 2p doublet was fitted with the following constraints: (i) spin–orbit splitting fixed at 1.18 eV (S 2p_3/2_–S 2p_1/2_), and (ii) area ratio of S 2p_1/2_:S 2p_3/2_ fixed at 0.50. Peak shapes were fitted using mixed Gaussian–Lorentzian functions (GL mix) with a common full width at half maximum (FWHM) within each chemical-state component. Reduced sulfur species were modeled near ~163–165 eV, and oxidized sulfur species were modeled near ~168–170 eV when present. Fit quality was evaluated by visual inspection of the overlay and by residual plots (measured spectrum minus fitted model), confirming the absence of systematic deviations across the fitted region. Each group was analyzed with at least three independently prepared samples (*n* ≥ 3).

### 2.5. Ellman’s Assay for Quantification of Surface-Accessible Thiol Groups

5,5′-Dithiobis(2-nitrobenzoic acid) (DTNB; Ellman’s reagent) ( Sigma-Aldrich, Merck KGaA, St. Louis, MO, USA), L-cysteine (Sigma-Aldrich/Merck, USA) as a thiol standard, and phosphate-buffered saline (PBS, pH 7.4) (Gibco, Thermo Fisher Scientific, Grand Island, NY, USA) were used. All reagents were of analytical grade. DTNB stock solution was prepared at 10 mM in PBS (pH 7.4) and protected from light. Immediately before use, DTNB stock was diluted to a working concentration of 0.5–1.0 mM in PBS. All DTNB-containing solutions were handled in amber tubes or wrapped in aluminum foil to minimize photodegradation. To quantify surface-accessible thiols, SP, SP-SH, and ALTUM powders were weighed (typically 5–10 mg per sample) and suspended in PBS (e.g., 500 µL^−1^ mL) in low-binding microcentrifuge tubes (Eppendorf SE, Hamburg, Germany). DTNB working solution was added to each suspension to reach the final DTNB concentration of 0.5–1.0 mM. Samples were incubated at room temperature for 30–60 min with gentle shaking (ThermoMixer, Eppendorf SE, Hamburg, Germany) to allow reaction of DTNB with accessible thiol groups, generating 2-nitro-5-thiobenzoate (TNB). After incubation, suspensions were centrifuged (e.g., 10,000× *g*, 5 min), and the supernatant was transferred to a 96-well plate (Corning Inc., Corning, NY, USA) for absorbance measurement. Absorbance at 412 nm was measured using a microplate spectrophotometer (SpectraMax iD5, Molecular Devices, LLC, San Jose, CA, USA) or equivalent. A standard curve was generated using L-cysteine standards prepared in PBS at multiple concentrations spanning the linear range (e.g., 0–100 µM). Sample thiol content was calculated from the standard curve and normalized to spicule mass (e.g., µmol SH equivalents per g of spicule). Each condition was measured in at least five replicates (*n* ≥ 5), and results were reported as mean ± standard deviation. Reduced glutathione (GSH) and oxidized glutathione (GSSG) were each used at 10 mM, reflecting the upper end of the physiological intracellular GSH range (1–10 mM) and matching the de facto standard concentration adopted in benchmarking disulfide-based redox-responsive carriers [10].

### 2.6. In Vitro Cumulative Release

To compare the release profiles of GHK-peptide from ALTUM and a physical mixture of sponge spicules with GHK (SP + GHK), an in vitro release study was performed using a dialysis-based method. ALTUM composite (10 mg), containing covalently conjugated GHK-peptide, was dispersed in 2 mL of phosphate-buffered saline (PBS). For comparison, a physical mixture consisting of sponge spicules and GHK-peptide (SP + GHK, total 10 mg) was prepared in 2 mL of PBS. Each formulation was transferred into a dialysis bag (molecular weight cut-off: 5 kDa), allowing the diffusion of released free GHK-peptide into the external medium while retaining the composite materials. The dialysis bags were immersed in 25 mL of PBS supplemented with 0.5% sodium dodecyl sulfate (SDS) to maintain sink conditions and facilitate peptide diffusion. The system was maintained at 37 °C under gentle agitation (30 rpm).

At predetermined time points (0.5, 1, 2, 4, 8, 12, 24, 48, and 72 h), 0.5 mL of the external release medium was collected and replaced with an equal volume of fresh medium. The collected samples were analyzed to quantify the released GHK-peptide. The concentration of GHK-peptide in the release medium was determined using UV–Vis spectrophotometry (GENESYS, Thermo Scientific, Thermo Fisher Scientific, Waltham, MA, USA) by measuring absorbance at 220 nm, corresponding to peptide bond absorption. A standard calibration curve was generated using known concentrations of GHK-peptide under identical buffer conditions. Background absorbance from PBS/SDS medium was subtracted prior to analysis. Cumulative release of GHK-peptide was calculated by correcting for sample withdrawal and medium replacement at each time point. The total amount of peptide released was expressed as a percentage of the initial GHK content loaded in each sample. Release profiles were plotted as cumulative percentage release versus time.

### 2.7. In Vitro Stability

To evaluate the stability of GHK-peptide conjugated within ALTUM under various environmental conditions, ALTUM samples were subjected to 4 °C, 45 °C, cyclic temperature changes (alternating between 4 °C and 45 °C every 12 h), and continuous visible light exposure over a 30-day period. Identical ALTUM samples were placed in PBS with 0.5% SDS, simulating physiological conditions. After 30 days, the GHK-peptide content in each sample was quantified by suspending ALTUM in PBS and measuring the peptide concentration using a UV-Vis spectrophotometer referred described methods. The percentage of GHK-peptide remaining was calculated by comparing the final peptide content to the initial loading.

### 2.8. Immunofluorescence Assay

To detect collagen protein in the artificial skin model, an immunofluorescence staining protocol was used. The model was first washed with PBS and fixed with 4% paraformaldehyde for 15–20 min, followed by permeabilization with 0.1% Triton X-100. After blocking with 5% BSA, the samples were incubated overnight at 4 °C with a primary antibody specific to collagen type I, diluted in PBS with 1% BSA. The next day, the samples were washed and then incubated with a fluorophore-conjugated secondary antibody, such as Alexa Fluor 488 or 594, for 1 h in the dark. After washing, the samples were optionally stained with DAPI to visualize nuclei, mounted on slides with anti-fade medium, and imaged using a fluorescence microscope. The fluorescence intensity of the collagen signal was quantified using image analysis software like ImageJ version 1.5, allowing for assessment of collagen levels within the artificial skin model and comparison across experimental conditions.

### 2.9. Western Blotting

To detect collagen, fibronectin, TGF-β, and SMAD proteins, samples were lysed with RIPA buffer containing protease and phosphatase inhibitors, and protein concentration was determined using a BCA assay. Equal amounts of protein (20–40 µg) were denatured and separated via SDS-PAGE, then transferred to a PVDF membrane. The membrane was blocked with 5% milk or BSA in TBST, followed by overnight incubation at 4 °C with primary antibodies specific to the target proteins. After washing, the membrane was incubated with an HRP-conjugated secondary antibody for 1 h, and protein bands were visualized using ECL and imaged. Band intensity was quantified with image analysis software and normalized to a loading control, providing precise detection and quantification of the target proteins.

### 2.10. Artificial Skin Preparation and Permeabilization Assay

Human foreskin fibroblasts (HFF-1) were cultured in Dulbecco’s Modified Eagle Medium (DMEM) supplemented with 15% fetal bovine serum (FBS) and 1% penicillin/streptomycin at 37 °C in a humidified atmosphere with 5% CO_2_. HaCaT keratinocytes were maintained under similar conditions in DMEM containing 10% FBS and 1% penicillin/streptomycin. For 3D bioprinting, dermal and epidermal cell populations were prepared using human-derived cells only. HFF-1 fibroblasts (1 × 10^7^ cells/mL) and HaCaT keratinocytes (2 × 10^7^ cells/mL) were suspended in a bioink composed of serum-free DMEM containing fibrinogen (20 mg/mL), gelatin (10% *w*/*v*), and sodium alginate (1% *w*/*v*). The mixture was homogenized to obtain a printable hydrogel. The bioink was loaded into a syringe fitted with a 22G nozzle and printed using a 3D bioprinter (Dr. Invivo; Rokit Healthcare, Seoul, Republic of Korea) onto Transwell inserts (12-well format, pore size: 3 μm). Constructs were fabricated in a cylindrical geometry (10 mm diameter, 3 mm height) at a dispensing pressure of 300 kPa and a filling density of 35%. Following printing, constructs were crosslinked using a solution containing calcium chloride and thrombin (20 U/mL) for 15 min to induce ionic crosslinking of alginate and enzymatic polymerization of fibrinogen. The constructs were then maintained under submerged culture conditions for 2 days in DMEM supplemented with 15% FBS to allow structural stabilization. Subsequently, constructs were transferred to air–liquid interface (ALI) culture conditions and maintained for 14 days in DMEM supplemented with 20% FBS, elevated calcium concentration (to promote keratinocyte differentiation), and keratinocyte growth supplements (HKGS), with medium changes every 2 days. This process enabled epidermal stratification and barrier formation, resulting in a multilayered human skin equivalent. For histological analysis, skin constructs were fixed in 4% formalin overnight, dehydrated using a tissue processor (TP1020; Leica, Deer Park, IL, USA), embedded in paraffin, and sectioned at 5 μm thickness using a microtome (RM2245, Leica Biosystems Nussloch GmbH, Nussloch, Germany). For cryosectioning, samples were cryoprotected in 10% sucrose for 1 h followed by 30% sucrose overnight, embedded in OCT compound, and frozen in liquid nitrogen. Sections (10 μm) were obtained using a cryostat (CM1860; Leica) at −24 °C. For permeabilization analysis, test formulations were applied uniformly to the surface of fully matured skin constructs. After defined incubation periods, samples were collected and sectioned for analysis. Penetration into epidermal and dermal layers was evaluated using fluorescence or confocal microscopy when labeled compounds were used. Quantitative analysis of penetration was performed by measuring signal intensity profiles along the tissue depth (*z*-axis), allowing comparison of penetration efficiency between experimental groups.

### 2.11. Data Analysis and Statics

Statistical analyses were performed using GraphPad Prism 8 (GraphPad Software). For in vitro experiments, at least *n* = 3 biological replicates were used per condition. Pairwise comparisons were conducted using Student’s *t*-tests, while multiple group comparisons were performed using one-way ANOVA followed by a post hoc test. Artificial skin experiments involved a minimum of *n* = 4 biological replicates per condition, with 2-group comparisons conducted using the non-parametric Mann–Whitney U test. No data pre-processing was applied prior to statistical analyses. Statistical significance was set at *p*-values below 0.05, with specific thresholds marked as * *p* < 0.05, ** *p* < 0.01, and *** *p* < 0.001. All quantitative data are presented as mean ± SD, and error bars together with significance markers (* *p* < 0.05, ** *p* < 0.01, *** *p* < 0.001) are displayed in the corresponding figures.

## 3. Results

### 3.1. Functionalization of Sponge Spicules and Covalent GHK-Tripeptide Conjugation

**Figure 1:**
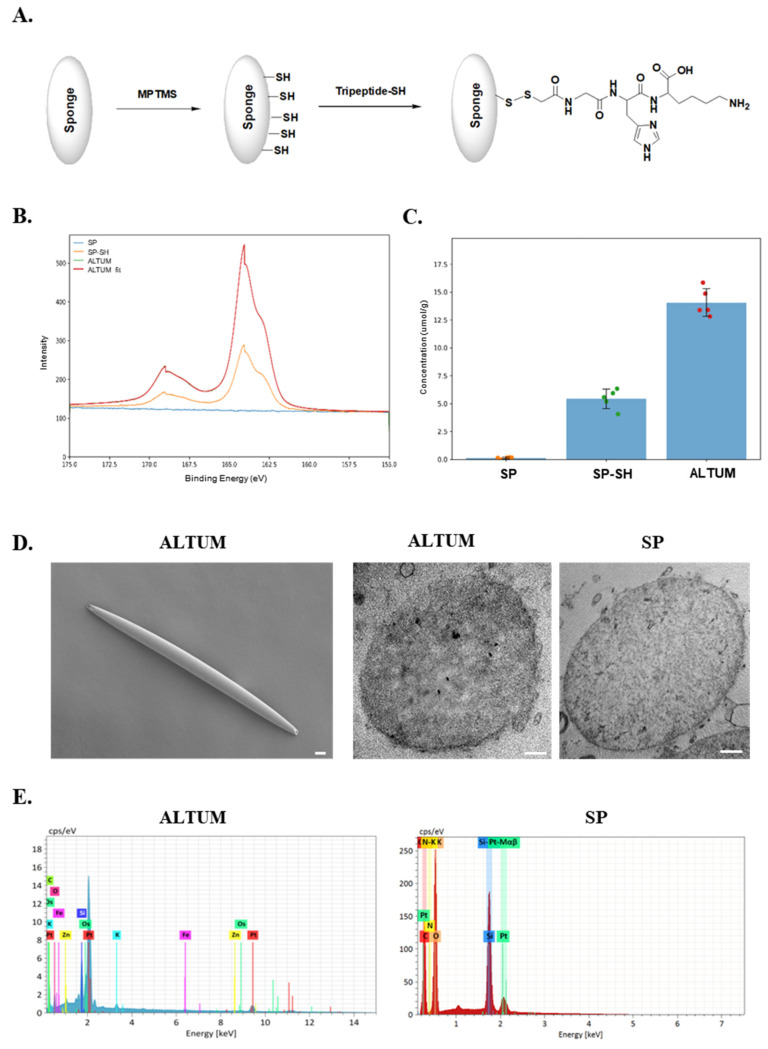
Surface thiol functionalization and GHK conjugation of sponge spicules. (**A**) Schematic of MPTMS-mediated thiol functionalization of sponge spicules (SP → SP-SH) and disulfide conjugation with HS-GHK to form ALTUM. (**B**) High-resolution XPS S 2p spectra of SP, SP-SH, and ALTUM. (**C**) Surface-accessible thiol quantification by Ellman’s assay (mean ± SD, *n* ≥ 5). (**D**) Representative SEM (ALTUM) and TEM (SP, ALTUM) images. (**E**) EDS spectra of SP and ALTUM.

Figure 1A illustrates the stepwise chemical modification of sponge spicules. Surface silanization with (3-mercaptopropyl)trimethoxysilane (MPTMS) introduced sulfhydryl (-SH) functionalities onto the spicule surface (SP-SH), followed by covalent conjugation with thiolated GHK peptide to generate ALTUM.

High-resolution S 2p spectra (Figure 1B) clearly demonstrate the successful introduction of sulfur-containing functionalities. Native SP exhibited no detectable S 2p signal, confirming the absence of intrinsic sulfur species. In contrast, SP-SH showed a characteristic S 2p doublet centered at approximately 164 eV, corresponding to reduced sulfur species introduced via MPTMS modification.

Importantly, ALTUM displayed a markedly increased S 2p intensity relative to SP-SH, indicating additional sulfur incorporation following GHK conjugation. Deconvolution of the ALTUM S 2p spectrum revealed a dominant reduced sulfur component (S 2p_3/2_ at 164.05 eV, FWHM ≈ 1.25 eV) and a minor oxidized sulfur contribution at ~169.00 eV. Peak fitting parameters are summarized in Supplementary Table S1. The constrained doublet splitting (1.18 eV) and area ratio (2p_1/2_:2p_3/2_ = 0.5) were applied during fitting, and the corresponding residual plot (Supplementary Figure S1) exhibited a random distribution around zero, supporting the robustness of the fitting model. Wide-scan XPS survey spectra (Supplementary Figure S2) further confirmed the presence of sulfur in SP-SH and ALTUM, while SP showed no S signal. Notably, ALTUM exhibited increased nitrogen (N 1s) intensity compared to SP-SH, consistent with the presence of peptide-derived amide groups on the surface. Collectively, these XPS results demonstrate successful thiol functionalization of sponge spicules and stable surface incorporation of sulfur-containing GHK moieties in ALTUM.

Surface-accessible thiol groups were quantified using Ellman’s assay (Figure 1C). Native SP exhibited negligible thiol signal, confirming the absence of free sulfhydryl groups. SP-SH showed a significant increase in thiol density, validating successful silanization. ALTUM exhibited a thiol-associated signal consistent with the sulfur presence detected by XPS. The comparable or enhanced thiol-related signal relative to SP-SH indicates that sulfur-containing functionalities remain accessible after peptide conjugation, supporting the successful surface presentation of GHK via thiol-mediated linkage.

SEM imaging confirmed that the characteristic needle-like morphology of sponge spicules was preserved after chemical modification (Figure 1D). TEM analysis further revealed subtle surface alterations in ALTUM compared to SP, consistent with the presence of a surface-bound organic layer.

Energy-dispersive X-ray spectroscopy (EDS) spectra (Figure 1E) showed the emergence of additional elemental signals in ALTUM compared to SP, including carbon and nitrogen species attributable to peptide incorporation. These findings further support the successful surface functionalization and conjugation process.

### 3.2. Redox-Responsive Cleavage-Driven Acceleration of GHK Release from ALTUM

**Figure 2:**
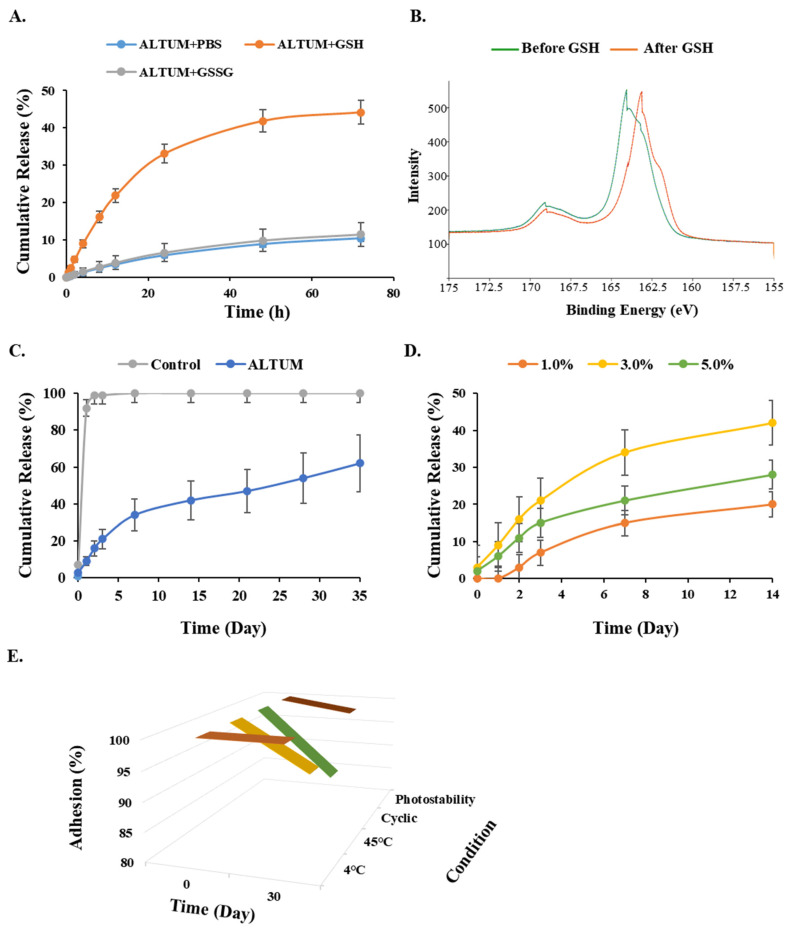
Redox-responsive and sustained release of GHK from ALTUM. (**A**) In vitro cumulative GHK release from ALTUM under PBS, GSSG (10 mM), and GSH (10 mM) conditions over 72 h; SP + GHK physical mixture is shown as control. (**B**) High-resolution XPS S 2p spectra of ALTUM before and after GSH treatment, showing reductive cleavage of the disulfide component. (**C**) Long-term cumulative release of GHK from ALTUM and SP + GHK control over 35 days in PBS. (**D**) Release profiles of ALTUM prepared at 1%, 3%, and 5% (*w*/*w*) GHK loading. (**E**) Thirty-day GHK retention in ALTUM under 4 °C, 45 °C, cyclic (4 °C/45 °C every 12 h), and visible-light (photostability) conditions. Data are mean ± SD.

To characterize how the disulfide linkage within ALTUM influences peptide release, cumulative release kinetics were first evaluated under reductive and oxidizing conditions (Figure 2A). In phosphate-buffered saline (PBS), ALTUM exhibited a slow and diffusion-limited release profile over 72 h, consistent with stable surface conjugation. Similarly, in the presence of oxidized glutathione (GSSG), the release kinetics remained comparable to baseline conditions, indicating that oxidizing environments do not significantly destabilize the conjugate. In contrast, exposure to reduced glutathione (GSH) resulted in a pronounced acceleration of GHK release. Under reductive conditions, ALTUM displayed a markedly increased initial release rate, with cumulative release rising rapidly within the first 24 h and reaching approximately four-fold levels of 72 h compared to both PBS and GSSG groups. The divergence in released profiles between GSH and GSSG conditions demonstrates that peptide detachment is reduction-specific rather than driven by nonspecific buffer effects.

To directly verify that GSH-induced acceleration arises from cleavage of disulfide linkages, high-resolution S 2p XPS analysis was performed before and after GSH treatment (Figure 2B). Prior to reduction, the S 2p spectrum was dominated by a disulfide-associated component centered near ~164 eV. Following GSH exposure, the relative intensity of the disulfide component decreased, accompanied by a corresponding increase in thiol/thiolate-related signals. This shift in sulfur chemical states confirms reductive cleavage of S-S bonds on the ALTUM surface and supports a cleavage-mediated release mechanism.

Having established reduction-triggered acceleration, we next evaluated the long-term release behavior of ALTUM under physiological conditions (Figure 2C). In contrast to the rapid depletion observed in the physical mixture control, ALTUM exhibited sustained peptide release over 35 days, gradually reaching approximately 60% cumulative release. These data indicate that, in the absence of reductive stimuli, the disulfide-mediated conjugation confers prolonged peptide retention. The incomplete release under non-reductive conditions reflects the intended design rather than peptide degradation: because release is governed by the stability of the disulfide tether, the retained (≈40%) fraction represents a peptide that remains covalently immobilized in the absence of a reductive trigger and constitutes a stable reservoir available for subsequent reduction-triggered release. This retention is functionally advantageous, as it limits premature, nonspecific loss and supports prolonged, controlled peptide availability at the application site.

Optimization of peptide loading ratios (1%, 3%, and 5% *w*/*w*) revealed similar sustained release patterns across formulations, with the 3% ALTUM condition providing balanced early-stage release and long-term retention (Figure 2D). This formulation was therefore selected for subsequent experiments. The 3% (*w*/*w*) ratio thus defines the peptide loading used throughout this study, and the availability of surface-accessible thiol groups for conjugation was confirmed by Ellman’s assay (Figure 1C).

To characterize the underlying release mechanism, the cumulative release profiles were fitted to the Korsmeyer–Peppas model (Mt/M∞ = k·t^n^), applied to the fractional release range below 60%. Under non-reductive conditions (PBS and GSSG), the fitted release exponent was n ≈ 0.35 (R^2^ > 0.97), corresponding to Fickian (diffusion-controlled) release consistent with stable surface tethering of GHK. Under reductive (GSH) conditions, the exponent increased toward n ≈ 0.47 with a substantially higher rate constant, indicating a shift toward anomalous (non-Fickian) transport in which diffusion is augmented by additional peptide detachment. These results indicate that the baseline release is diffusion-controlled, whereas the accelerated release under reductive conditions is not governed by diffusion alone.

Finally, structural stability of ALTUM was evaluated under multiple storage conditions, including 4 °C, 45 °C, cyclic temperature variation, and photostability (Figure 2E). Across all conditions, minimal peptide loss was observed over 30 days, indicating that the conjugate remains stable under environmental stress in the absence of reductive triggers.

Collectively, these findings demonstrate that ALTUM functions as a sustained, controlled-release delivery system: the disulfide linkage ensures prolonged peptide retention under physiological and storage conditions, while release of GHK is accelerated in reductive (GSH-containing) environments. This GSH-dependent release behavior supports the potential of ALTUM as a controlled-release platform for dermal peptide delivery, with the underlying redox-triggered mechanism discussed below.

### 3.3. Effective GHK-Peptide Delivery into Artificial Human Skin via ALTUM

**Figure 3:**
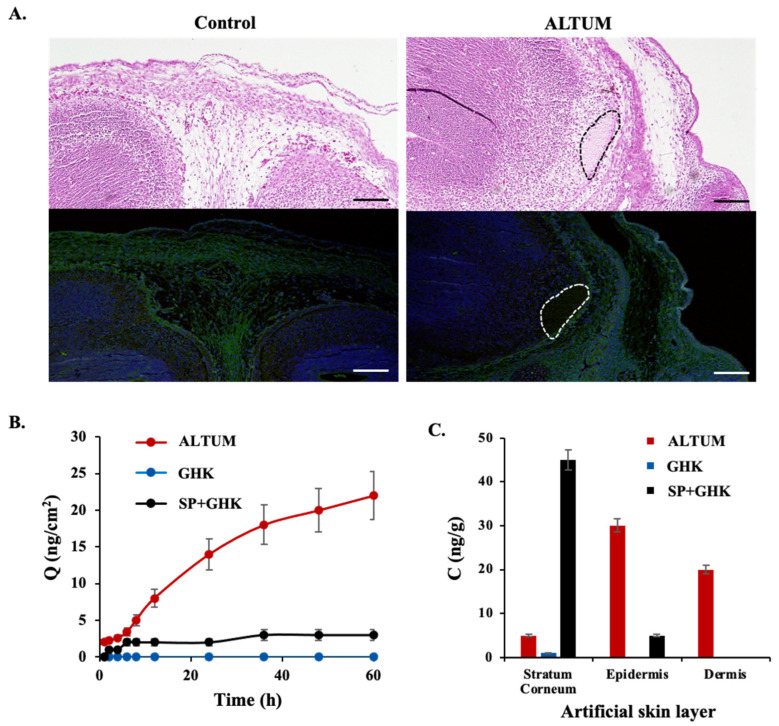
Dermal delivery of GHK by ALTUM in a bioprinted artificial human skin model. (**A**) Representative immunofluorescence images showing ALTUM penetration into bioprinted skin constructs. Green: collagen; blue: DAPI; white dotted line: ALTUM. Scale bar = 500 µm. (**B**) Quantitative GHK content in skin constructs treated with free GHK, SP + GHK, or ALTUM. (**C**) GHK distribution across histological skin layers. Data are mean ± SD.

Figure 3A explores the efficacy of GHK-peptide delivery into a bioprinted artificial human skin model using ALTUM, with a focus on histological analysis. The artificial human skin was created by culturing human skin cell lines in a collagen-alginate matrix over five weeks, resulting in a well-organized structure that closely mimics the epidermal and dermal layers of human skin (Control), as indicated by the green-stained collagen. Upon gentle application of ALTUM to the surface of the artificial skin, ALTUM particles were observed to successfully traverse the epidermal barrier and distribute within the underlying tissue layers (marked by dotted lines).Three reference conditions were compared in parallel to dissect the contributions of the spicule scaffold and the conjugation chemistry: free GHK alone (cargo-only), SP + GHK physical mixture (scaffold-plus-cargo without covalent tethering), and ALTUM (covalently disulfide-tethered). In addition, thiolated spicules without conjugated peptide (SP-SH), characterized in Figure 1B,C, served as a no-peptide control; the absence of GHK in SP-SH accounts for its lack of collagen-inducing activity, thereby isolating the contribution of the conjugated peptide. Conversely, because native spicules (SP) lack surface sulfhydryl groups (confirmed by XPS and Ellman’s assay), the SP + GHK physical mixture also serves as a non-thiolated reference in which covalent disulfide tethering cannot occur; the marked functional difference between SP + GHK and ALTUM therefore reflects the importance of thiol-mediated covalent conjugation. To quantify transdermal GHK delivery, GHK-peptide concentrations within the separated artificial skin layers were extracted and measured using UV-Vis spectrophotometry at 220 nm. While free GHK and the physical mixture (SP + GHK) showed limited penetration, reaching an average concentration of 1.9 ng/cm^2^, ALTUM facilitated a more than fivefold increase in delivery, achieving 9.72 ng/cm^2^. This significant enhancement is suggested to be facilitated by the unique morphology of the spicules (Figure 1D), which appears to enhance delivery across the outer skin barriers. Furthermore, the sustained delivery profile observed over 60 h (Figure 3B) is consistent with the stable retention of the peptide via disulfide linkage, potentially preventing rapid premature release before reaching the target layers. This enhanced distribution into deeper layers (Figure 3C) represents a substantial improvement in delivery efficiency compared to the conventional mixture (Figure 3B).

Further analysis comparing the distribution of GHK-peptide across different skin layers confirmed ALTUM’s superior penetration capabilities. While the SP + GHK group showed GHK-peptide primarily localized to the outermost stratum corneum with minimal presence in the epidermis, the ALTUM-treated group exhibited substantial peptide presence not only in the epidermis but also extending into underlying tissue layers (Figure 3C). This indicates a significant improvement in transdermal delivery efficiency and depth of penetration when using ALTUM compared to the conventional mixture of SP and GHK-peptide.

### 3.4. ALTUM Enhances Collagen Formation in Artificial Human Skin and Fibroblast Cells

**Figure 4:**
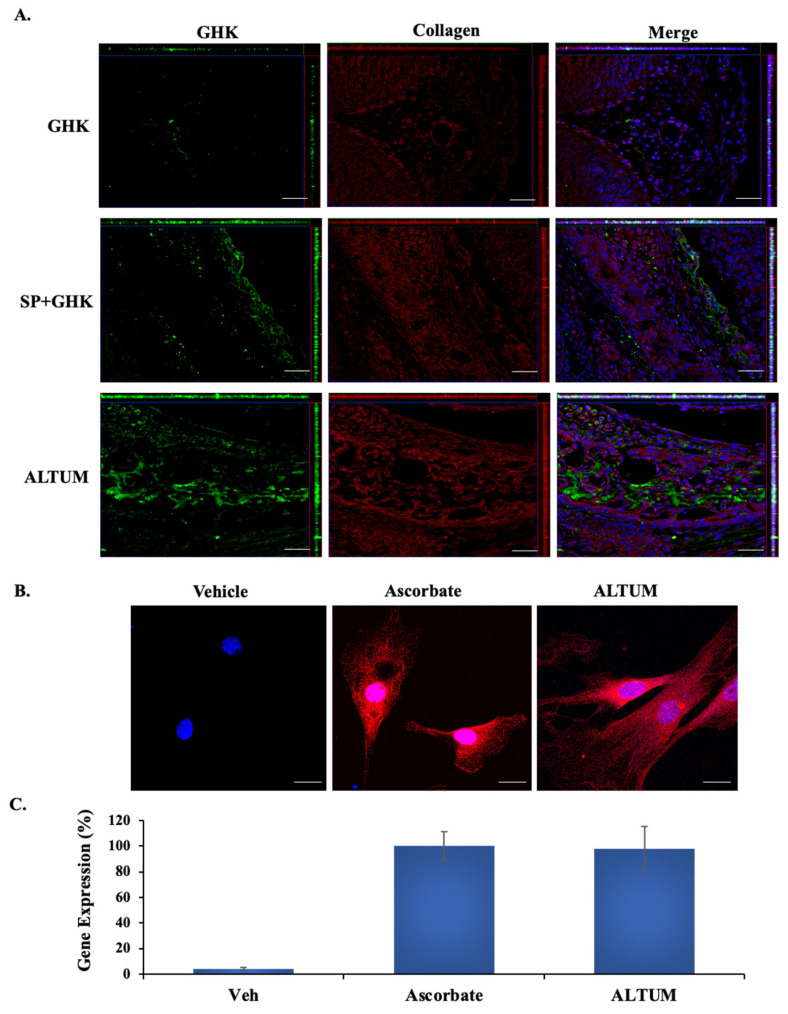
ALTUM-induced collagen expression in artificial skin and human fibroblasts. (**A**) Immunofluorescence of collagen (red) and GHK (green) in artificial skin treated with GHK, SP + GHK, or ALTUM. Scale bar = 100 µm. (**B**) Collagen immunofluorescence in HFF-1 fibroblasts treated with ALTUM or ascorbic acid (positive control). Scale bar = 10 µm. (**C**) RT-qPCR analysis of COL1A1 expression in HFF-1 fibroblasts. Data are mean ± SD.

The GHK-peptide has been extensively reported to promote collagen synthesis, a critical function in wound healing and skin regeneration. In our study, we compared the collagen formation abilities of ALTUM, SP + GHK, and GHK-peptide alone in artificial human skin. The results demonstrated that ALTUM significantly enhanced the production of Type I collagen, the most abundant structural protein in the dermis. Notably, while Type I collagen levels were markedly increased, the expression of other structural proteins such as fibronectin and laminin remained relatively unchanged. This selective pattern of protein expression suggests a targeted response rather than a generalized upregulation of ECM components, consistent with the known specificity of GHK peptide for collagen-related processes as reported in previous studies (Figure 4A).

Further assessment of collagen formation was performed using human fibroblast cells in vitro (Figure 4B). Immunofluorescence staining indicated that ALTUM induced collagen protein levels comparable to those observed in the positive control group treated with ascorbic acid, a well-known stimulator of collagen synthesis. Additionally, RT-qPCR analysis of the same in vitro model confirmed that ALTUM and ascorbic acid both resulted in similar levels of collagen gene expression, underscoring the potent collagen-inducing effects of ALTUM at the molecular level (Figure 4C).

### 3.5. Mechanistic Insights into Collagen Formation Induced by ALTUM in Fibroblast Cells

**Figure 5:**
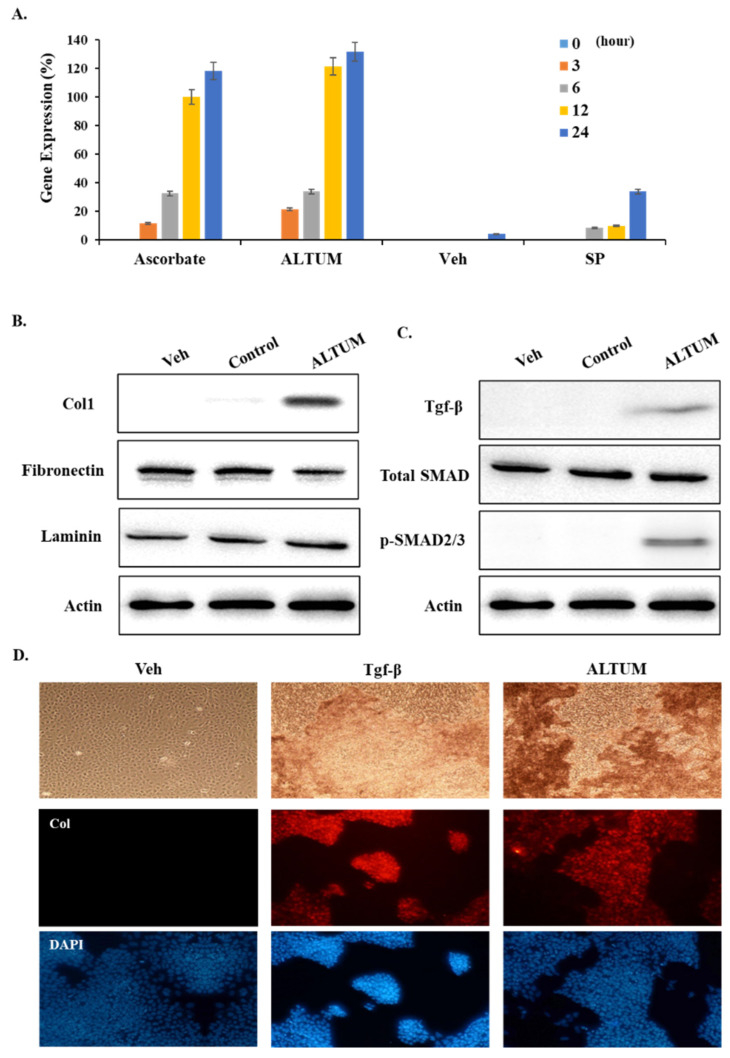
Engagement of the TGF-β–SMAD axis in ALTUM-treated fibroblasts. (**A**) Time course of COL1A1 expression over 24 h post-treatment (RT-qPCR). (**B**,**C**) Western blot analysis of fibronectin, laminin, TGF-β, total SMAD2/3, and phosphorylated SMAD2/3 in HFF-1 fibroblasts at 24 h post-treatment, with band-intensity quantification normalized to loading control. (**D**) Immunofluorescence of collagen fibrous structures following ALTUM treatment. Red: collagen; blue: DAPI. Data are mean ± SD.

The precise mechanisms underlying the collagen-promoting effects of GHK-peptide remain incompletely understood. However, our in vitro studies with human fibroblast cells reveal that ALTUM mirrors the collagen synthesis timeline observed with ascorbic acid, a well-known inducer of collagen production (Figure 5A). Previous studies have shown that ascorbic acid begins to enhance collagen synthesis approximately 12 h post-treatment, a pattern also observed with ALTUM, which significantly increased collagen production after the same duration.

Interestingly, ALTUM specifically enhances collagen synthesis without affecting the expression of other structural proteins such as fibronectin or laminin (Figure 5B). This selective upregulation suggests that ALTUM’s effect is highly targeted towards the collagen production pathway, distinguishing it from other extracellular matrix proteins.

Further mechanistic analysis demonstrated that ALTUM treatment led to an upregulation of TGF-β expression in fibroblast cells, a key growth factor involved in collagen synthesis. This upregulation was accompanied by a significant increase in the phosphorylated form of SMAD2/3 proteins, despite no changes in total SMAD levels (Figure 5C). These observations are consistent with engagement of the TGF-β–SMAD signaling axis following ALTUM treatment, although the present data are correlative and do not establish that this pathway is strictly required for the observed collagen induction.

Moreover, when TGF-β was directly applied to cultured fibroblast cells, a marked increase in collagen synthesis was observed, paralleling the effects seen with ALTUM treatment (Figure 5D). This suggests that ALTUM may exert its collagen-promoting effects at least in part by enhancing TGF-β signaling, leading to the activation of downstream pathways that specifically drive collagen production.

## 4. Discussion

In this study, we developed a thiol-functionalized sponge spicule composite, ALTUM, designed for controlled dermal delivery of GHK peptide and enhanced collagen synthesis. By covalently conjugating GHK to sulfhydryl-modified spicules via disulfide linkage, ALTUM achieved stable surface presentation and sustained peptide retention under physiological and storage conditions. Importantly, this conjugation strategy also conferred redox-responsive behavior, whereby exposure to reduced glutathione (GSH) selectively accelerated peptide release through disulfide bond cleavage. This dual release profile—long-term stability with reduction-triggered activation, distinguishes ALTUM from conventional delivery systems. Functionally, ALTUM significantly improved transdermal penetration in an artificial human skin model and enhanced collagen production in dermal fibroblasts. Mechanistically, ALTUM treatment correlated with increased TGF-β/SMAD pathway markers, as evidenced by increased phosphorylation of SMAD2/3 without changes in total SMAD levels. Collectively, these findings establish ALTUM as a structurally stable yet stimuli-responsive platform that integrates controlled release with targeted bioactivity to promote collagen synthesis in dermal tissue.

Previous studies have explored various approaches to functionalizing sponge spicules for biomedical applications [11]. For example, some researchers have functionalized sponge spicules with amine groups to enhance their interaction with bioactive molecules [12], while others have employed cationic liposome techniques to create a more reactive surface for subsequent conjugation with therapeutic agents [13,14]. However, these spicule-based systems rely on physical mixing or non-cleavable conjugation, providing little control over post-penetration release kinetics. In parallel, disulfide-based redox-responsive carriers—most notably mesoporous silica nanoparticles capped with GSH-cleavable gatekeepers—have been extensively developed for intracellular drug release in oncology applications [14,15], but these systems were not designed for transdermal use and lack the mechanical penetration capability required for dermal targeting. Conversely, GHK delivery systems based on liposomes or photo-crosslinkable HA hydrogels embedding Cu-GHK nanofibers have advanced GHK bioavailability [7,16], yet remain limited by passive diffusion across the stratum corneum and the absence of stimulus-responsive control of release. ALTUM bridges these previously separate design strategies by integrating, within a single composite, (i) spicule-mediated mechanical penetration, (ii) covalent disulfide tethering for sustained retention under storage and physiological conditions, and (iii) GSH-triggered acceleration of GHK release within the reductive dermal microenvironment. The comparative positioning of ALTUM relative to representative prior systems is summarized in Table 1.

The accelerated GHK release observed under reductive conditions is consistent with a redox-triggered release mechanism. Because the disulfide (S–S) bond is the only labile linkage introduced during conjugation, GSH is expected to cleave this bond through thiol–disulfide exchange, thereby releasing the tethered peptide. The substantially higher intracellular GSH concentration (1–10 mM) relative to the extracellular environment (2–20 µM) provides a plausible physiological basis for preferential release within the reductive dermal microenvironment. While the present release and kinetic data are fully consistent with this proposed redox-triggered mechanism, direct molecular confirmation of the released species (e.g., by LC-MS or HPLC) remains to be performed and is identified as a near-term extension of this work. With respect to whether the in vitro GSH concentration realistically mimics the dermal environment, it should be noted that disulfide cleavage occurs predominantly within the reductive intracellular compartment encountered by the peptide after cellular uptake, rather than in the extracellular space; the 10 mM condition is therefore intended to model this intracellular environment, which falls within the reported 1–10 mM range for dermal fibroblasts and keratinocytes. Intracellular GSH is moreover frequently elevated in proliferative and wound-healing dermal states, suggesting that the redox-triggered behavior of ALTUM may be especially relevant in regenerative contexts. Direct measurement of dermal GSH in the target tissue was not performed here and represents a useful direction for future validation.

Sponge spicule-based delivery systems have previously been reported to exhibit favorable biocompatibility, with mechanical skin disruption being reversible and well tolerated at therapeutically relevant doses [5,11,13]. Consistent with this, the bioprinted skin constructs used in the present study maintained intact epidermal stratification and continued collagen deposition following ALTUM application, indicating that the formulation does not grossly compromise tissue integrity under the conditions tested. As a marine sponge-derived biomaterial, the immunogenicity and inflammatory potential of the spicules also warrant consideration. The spicules used here are silica-based and were subjected to sequential NaOH and HCl treatment to remove organic and proteinaceous residues that could otherwise contribute to immune recognition, and purified silica spicules are generally regarded as biocompatible. Nevertheless, particulate silica and mechanically penetrating microstructures can in principle elicit local inflammatory responses; dedicated evaluation of cytokine release, complement activation, and local tissue reaction therefore represents an important prerequisite for clinical translation and is identified as future work.

The enhanced dermal delivery of GHK by ALTUM is attributed primarily to the needle-shaped morphology of the sponge spicules, which mechanically generates transient microchannels in the skin barrier and thereby facilitates passive penetration of the conjugated peptide into the dermis. This is a passive, physical mechanism rather than an active (energy-dependent) transport process. In addition, covalent tethering of GHK to the spicule surface promotes local retention and sustained, controlled release at the application site, in contrast to the rapid diffusional loss expected for free peptide. The combination of spicule-mediated microchannel formation (enhanced penetration) and retention-enhanced sustained release therefore accounts for the more than five-fold improvement in delivery relative to the physical mixture, with no evidence implicating active transport. However, these methods often resulted in composites with limited stability or inconsistent bioactivity, particularly under physiological conditions. In contrast, the functionalization of sponge spicules with sulfhydryl groups, followed by the covalent conjugation of GHK-peptide, as presented in this study, proved to be a more effective strategy for creating a stable and bioactive composite, ALTUM. The morphological analysis through SEM and TEM confirmed significant structural changes, indicative of successful peptide conjugation. The presence of diverse elements on the ALTUM surface, as revealed by EDS analysis, further validated the efficient functionalization. These findings suggest that the covalent bonding between GHK-peptide and the functionalized spicules is robust, providing a stable matrix for subsequent biological applications. The introduction of multiple sulfhydryl groups on the spicule surface not only facilitated the covalent attachment of GHK-peptide but also likely contributed to the sustained release observed in subsequent studies.

Importantly, beyond sustained release under physiological and storage conditions, ALTUM demonstrated redox-responsive behavior characterized by glutathione (GSH)-triggered acceleration of peptide release [15]. While ALTUM maintained a controlled release profile in PBS and in the presence of oxidized glutathione (GSSG), exposure to reduced GSH markedly increased the cumulative release rate within 72 h. High-resolution S 2p XPS analysis further confirmed that GSH treatment altered sulfur chemical states, consistent with reductive cleavage of disulfide linkages on the ALTUM surface [16]. This reduction-specific response indicates that peptide detachment is mediated by disulfide bond cleavage rather than nonspecific environmental effects. Such redox-responsive behavior is particularly relevant in cutaneous tissues, where intracellular and microenvironmental glutathione levels can provide a reductive milieu [17]. Therefore, ALTUM uniquely combines long-term stability with stimuli-responsive release, enabling stable peptide retention under storage conditions while facilitating accelerated release within reductive biological environments.

The release profile of GHK-peptide from ALTUM over 35 days demonstrated a sustained and controlled release, which marks a significant advancement compared to previous studies involving functionalized sponge spicules. In many earlier approaches, functionalization methods often resulted in a much faster release of bioactive agents, typically within just a few days, limiting their effectiveness for long-term therapeutic applications [14,18]. In contrast, ALTUM exhibited a gradual release, reaching up to 60% of the conjugated peptide over 35 days, which is particularly advantageous for sustained therapeutic effects. Additionally, the remarkable stability of ALTUM under various environmental conditions, including different temperatures and light exposure, further highlights its potential for use in diverse biomedical settings. The minimal loss of GHK-peptide under these conditions, including the confirmed 30-day stability in cyclic temperature tests, demonstrates that ALTUM retains its conjugated peptide under thermal, cyclic, and photostability stressors over the tested 30-day period. Extended storage studies will be required to determine whether this stability profile is maintained over longer timeframes relevant to chronic therapeutic applications.

ALTUM’s ability to deliver GHK-peptide into artificial human skin more effectively than other methods highlights its potential as a superior transdermal delivery system. The successful penetration of GHK-peptide into the epidermal layer, as observed in the ALTUM-treated group, suggests that the spicule structure not only facilitates peptide delivery but also protects the peptide from degradation or premature release. The sustained presence of GHK-peptide within the skin layers, with a peak release observed at 36 h, further supports ALTUM’s efficacy in delivering bioactive peptides for skin regeneration applications, maintaining therapeutic levels for over 60 h. The comparison with other delivery methods, such as GHK-peptide alone or mixed with sponge spicules, clearly demonstrated ALTUM’s superiority in both delivery efficiency and penetration depth.

ALTUM significantly enhances collagen formation in both artificial human skin and fibroblast cells, aligning with the known bioactivity of GHK-peptide in promoting collagen synthesis. Immunofluorescence and RT-qPCR results demonstrated that ALTUM boosts collagen production to levels comparable to those induced by ascorbic acid, a well-established collagen stimulator. The dense collagen presence in the dermal layer of ALTUM-treated skin models suggests effective deposition in deeper skin layers, crucial for tissue regeneration and relevant to wound healing and anti-aging applications.

The upregulation of TGF-β and activation of the SMAD signaling pathway in ALTUM-treated fibroblast cells provides a mechanistic basis for the observed increase in collagen synthesis [19]. The specific rise in phosphorylated SMAD2/3 levels, without changes in total SMAD, indicates that ALTUM selectively activates the signaling pathways necessary for collagen gene transcription [20]. These findings suggest that ALTUM may enhance natural growth factor signaling, offering potential for targeted therapeutic applications. The selective upregulation of Type I collagen suggests a targeted remodeling process rather than a nonspecific fibrotic response. This selectivity likely reflects the dominant role of the TGF-β/SMAD axis in dermal fibroblasts, which primarily drives collagen gene transcription. Unlike conventional systems that may cause broad ECM accumulation, ALTUM’s sustained GHK release appears specifically optimized for triggering this regenerative pathway.

Limitations: While our findings support the involvement of the TGF-β–SMAD signaling axis in ALTUM-induced collagen synthesis, several limitations should be acknowledged. Foremost among these, the mechanistic evidence presented here is correlative: increased TGF-β expression and SMAD2/3 phosphorylation, together with the parallel response to exogenous TGF-β, are consistent with pathway engagement but do not formally establish that the TGF-β–SMAD axis is required for the observed collagen response. Definitive causality will require pharmacological inhibition (e.g., TGFβRI inhibitor SB431542) or genetic perturbation (e.g., SMAD2/3 siRNA or CRISPR-based knockdown) in future studies. Additional contributions from non-canonical or parallel signaling routes—including SMAD-independent TGF-β branches, BMP/SMAD1/5/8, or growth-factor crosstalk—also cannot be excluded based on the current dataset. In addition, UV–Vis spectrophotometry at 220 nm enables quantitative comparison among release conditions but does not confirm the molecular identity of the released species. Because the disulfide tether is the only labile bond introduced during conjugation, GSH-mediated cleavage is chemically expected to release intact HS-GHK; direct verification by LC-MS or HPLC is identified as an essential near-term extension of this work. Finally, two additional controls would refine mechanistic attribution beyond the existing SP + GHK, GSSG, and free-GHK references: (i) a non-cleavable linker control (thioether- or amide-tethered SP–GHK) to isolate the specific contribution of the disulfide bond, and (ii) a sustained free-peptide release control matched to ALTUM kinetics to distinguish release-kinetic effects from spicule-mediated penetration. These are identified as priority extensions of this work.

## 5. Conclusions

In this study, we developed ALTUM, a sulfhydryl-functionalized sponge spicule composite in which GHK peptide is covalently tethered through a disulfide linkage. ALTUM exhibited sustained peptide retention under physiological and storage conditions and accelerated GHK release in the presence of reduced glutathione (GSH), consistent with a redox-responsive release mechanism. In a bioprinted artificial human skin model, ALTUM showed greater dermal penetration and higher collagen deposition compared with a physical mixture of spicules and free GHK. Mechanistically, ALTUM-mediated delivery was associated with increased TGF-β expression and engagement of SMAD signaling, as evidenced by elevated phosphorylated SMAD2/3 levels, consistent with involvement of the TGF-β–SMAD axis in the observed collagen response—although formal pathway dependence remains to be established in future loss-of-function studies. Collectively, these findings establish ALTUM as a redox-sensitive dermal delivery platform that couples structural robustness with reduction-specific peptide release, offering a promising strategy for controlled skin regeneration therapeutics.

## Figures and Tables

**Table 1:** Comparative positioning of ALTUM relative to representative prior dermal/transdermal delivery systems.

System (Representative Reference)	Carrier Architecture	Conjugation Chemistry	Mechanical Penetration	Stimulus-Responsive Release	Cargo	Application Context
SHS spicules (Zhang et al., 2021 [11])	Marine *Haliclona* sp. spicules	Physical mixture	Yes (aspect-ratio-dependent)	No	Insulin/Cyclosporine A	Transdermal protein delivery
Cationic liposome–spicule (Liang et al., 2020 [13])	Spicule + cationic liposome	Electrostatic	Yes	No	siRNA	Skin gene delivery
MSN-SS-RGD (Ref. [15])	Mesoporous silica nanoparticle	Disulfide (S–S) gatekeeper	No	Yes (GSH)	Doxorubicin	Intracellular cancer therapy
Cu-GHK NF / HA-Ty hydrogel (Lee et al., 2023 [7])	Photo-crosslinked HA hydrogel	Self-assembled Cu-GHK nanofibers	No	No	Cu-GHK	Wound healing
GHK-Cu liposomes (Pickart et al., 2018 [16])	Lipid bilayer vesicle	Encapsulation	No	No	GHK-Cu	Topical anti-aging
**ALTUM (this study)**	Sulfhydryl-functionalized *Spongilla fragilis* spicule	Covalent disulfide (S–S) tether	Yes	Yes (GSH-triggered)	GHK tripeptide	Dermal collagen induction

## Data Availability

The data supporting the findings of this study are available within the article and its Supplementary Information files. The datasets generated and/or analyzed during the current study are available from the corresponding author upon reasonable request.

## Supplementary Materials

The following supporting information can be downloaded at: https://www.mdpi.com/article/10.3390/mi17060750/s1.
