# Supplementary material for: Redox-Responsive GHK-Conjugated Sponge Spicules for Sustained Dermal Delivery and Enhanced Collagen Synthesis
Source: Micromachines (Basel). 2026 Jun 21;17(6):750. doi: 10.3390/mi17060750 (PMC13302783; doi:10.3390/mi17060750)
Supplement: Supplementary file 1 [file micromachines-17-00750-s001.zip › micromachines-4311629-supplementary.pdf]

Supplementary Figure S1

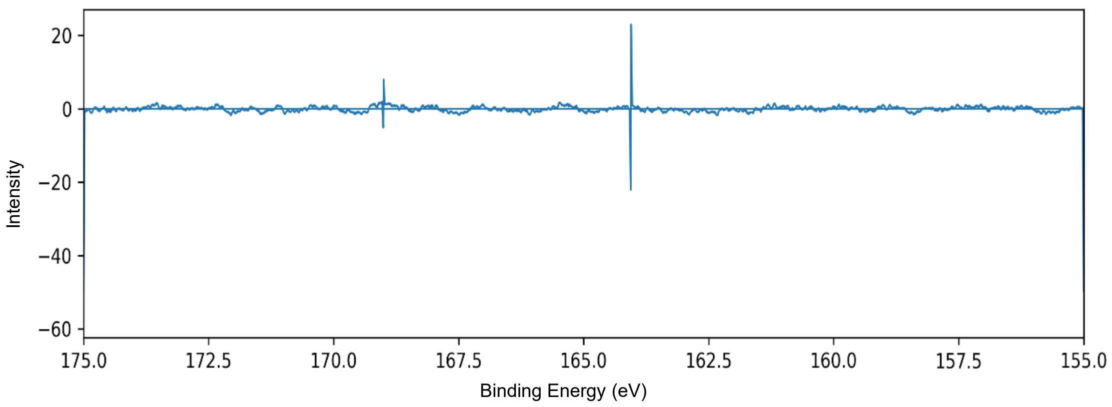

Supplementary Table S1

Peak component	S 2p3/2 center (eV)	FWHM (eV)	Doublet split (eV)	Area ratio (2p1/2:2p3/2)	Area (%)
Reduced S	164.05	1.25	1.18	0.50	80.3
Oxidized S	169.00	1.55	1.18	0.50	19.7

Supplementary Figure S2

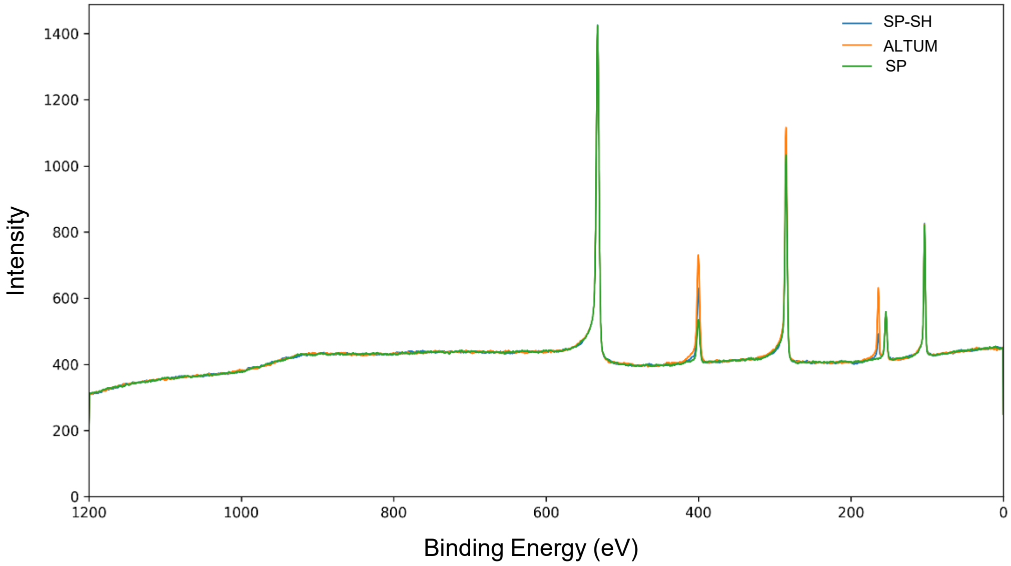

Peak (assignment)	Binding energy (eV)	Atom
Si 2p	103.3	Si
Si 2s	154.0	Si
S 2p	163.6–164.8	S
C 1s	285.0	C
N 1s	400.0	N
O 1s	532.2	O
